# Potential Reduction of Contralateral Second Breast-Cancer Risks by Prophylactic Mammary Irradiation: Validation in a Breast-Cancer-Prone Mouse Model

**DOI:** 10.1371/journal.pone.0085795

**Published:** 2013-12-20

**Authors:** Igor Shuryak, Lubomir B. Smilenov, Norman J. Kleiman, David J. Brenner

**Affiliations:** Center for Radiological Research, Columbia University Medical Center, New York, New York, United States of America; IIT Research Institute, United States of America

## Abstract

**Background:**

Long-term breast-cancer survivors have a highly elevated risk (1 in 6 at 20 years) of contralateral second breast cancer. This high risk is associated with the presence of multiple pre-malignant cell clones in the contralateral breast at the time of primary breast cancer diagnosis. Mechanistic analyses suggest that a moderate dose of X-rays to the contralateral breast can kill these pre-malignant clones such that, at an appropriate Prophylactic Mammary Irradiation (PMI) dose, the long-term contralateral breast cancer risk in breast cancer survivors would be considerably decreased.

**Aims:**

To test the predicted relationship between PMI dose and cancer risk in mammary glands that have a high risk of developing malignancies.

**Methods:**

We tested the PMI concept using MMTV-PyVT mammary-tumor-prone mice. Mammary glands on one side of each mouse were irradiated with X-rays, while those on the other side were shielded from radiation. The unshielded mammary glands received doses of 0, 4, 8, 12 and 16Gy in 4-Gy fractions.

**Results:**

In high-risk mammary glands exposed to radiation doses designed for PMI (12 and 16 Gy), tumor incidence rates were respectively decreased by a factor of 2.2 (95% CI, 1.1-5.0) at 12 Gy, and a factor of 3.1 (95% CI, 1.3-8.3) at 16 Gy, compared to those in the shielded glands that were exposed to very low radiation doses. The same pattern was seen for PMI-exposed mammary glands relative to zero-dose controls.

**Conclusions:**

The pattern of cancer risk reduction by PMI was consistent with mechanistic predictions. Contralateral breast PMI may thus have promise as a spatially targeted breast-conserving option for reducing the current high risk of contralateral second breast cancers. For estrogen-receptor positive primary tumors, PMI might optimally be used concomitantly with systemically delivered chemopreventive drugs such as tamoxifen or aromatase inhibitors, while for estrogen-receptor negative tumors, PMI might be used alone.

## Introduction

Breast cancer is one of the most common types of cancer in the US, and as many as one in eight women may develop breast cancer during their lifetime [1]. Long term survival after breast cancer diagnosis has increased markedly in the last decade, with the mean 15-year relative survival in the US now 77% [1]. It is therefore highly appropriate that increasing attention is being paid to the issue of breast cancer survivorship and to the issue of second breast cancers [2]. In particular, long term studies suggest that a 20-year breast cancer survivor has about a 1 in 6 risk of developing a contralateral second breast cancer [3,4], which is several-fold higher than primary breast cancer incidence among age-matched healthy women [5,6]. As a result of these long-term risks, between 10% and 20% of all breast cancer patients in the US currently undergo prophylactic contralateral mastectomy [7-9]. 

For estrogen-receptor positive tumors, adjuvant tamoxifen reduces contralateral breast cancer risks by as much as 40% [10] - which for long-term survivors represents a risk reduction from about 16% to 10%, still a disturbingly high risk. Tamoxifen is not considered effective for the ~30% of breast cancers which are estrogen-receptor negative [6,11,12]. A further incremental improvement for postmenopausal women is likely obtainable with aromatase inhibitor drugs [13], though again largely for estrogen-receptor positive primary cancers [12]. Both classes of chemopreventive drugs have significant side effects: gynecological and thromboembolic in the case of tamoxifen, and reduced bone mineral density in the case of aromatase inhibitors. In summary, there is a need for alternative approaches to further reduce the long term risks of contralateral breast cancer in breast cancer survivors.

The general concept behind chemopreventive approaches is that carcinogenesis is a multi-stage process involving the gradual accumulation of pre-malignant clones, with chemopreventive drugs intervening at one or more stages in this process [14]. We have previously proposed [15], and here we provide some experimental validation for, an alternative (or concomitant) approach for reducing contralateral breast cancer risks – though with the same overall rationale, to intervene in the multi-stage processes leading here to contralateral breast cancer. Specifically, an intermediate dose of ionizing radiation, which is an effective and spatially targeted cytotoxic agent, has the potential to kill essentially all the pre-existing pre-malignant cells in the contralateral breast of a breast cancer patient, and thus significantly reduce the incidence of contralateral breast cancers. In such an approach it is not necessary to identify these pre-existing pre-malignant cells, in that radiation-induced cell killing is largely a statistical process: Assuming that these pre-existing pre-malignant cells are far fewer in number than the healthy human cells, which will always be the case, the suggestion is that they can be essentially all killed with a moderate dose of radiation that produces minimal normal-tissue complications [15].

Of course ionizing radiation is also carcinogenic, and can induce new breast tumors [16,17].The net effect, therefore, of irradiating a breast containing significant numbers of pre-existing pre-malignant clones (such as the contralateral breast of a breast-cancer patient) will be dominated by the balance of two opposing processes: 1) killing of pre-existing pre-malignant cell clones, leading to reduced background breast cancer risk, versus 2) net induction of new pre-malignant cell clones, leading to radiation carcinogenesis. One of the main rationales behind the current work is that the risk of radiation-induced breast-cancer decreases sharply with age at irradiation [16-18], becoming very low for women aged >50, who constitute the majority of breast cancer patients [17,18]. This observation is consistent with findings that breast cancer radiotherapy does not appear to increase the net risk of new (genetically independent of the primary tumor) breast cancers in the irradiated ipsilateral (affected) breast [17,19], and may even decrease it [20,21]. 

In fact, a mechanistically-based analysis [15] of the time-dependence of new (non-recurrent) breast cancers in the ipsilateral breast suggests that, even at the comparatively high radiotherapeutic doses generally used for breast-cancer radiotherapy (50 Gy in 2 Gy fractions to the entire affected breast), net induction of pre-malignant cells is essentially neutral: most pre-existing pre-malignant cells are killed, and a similar number of new ones are induced. 

The ipsilateral breast requires a high radiation dose in order to eradicate large numbers of remaining primary tumor cells. However the reasoning here suggests [15] that a much lower radiation dose would still be sufficient to kill the smaller number pre-existing pre-malignant cells, which are independent of the primary tumor – and of course a much lower radiation dose would be associated with much lower risks for inducing new cancers or normal-tissue complications. Thus, irradiating the *contralateral* breast of breast cancer patients with such a moderate radiation dose might be an effective option for safely reducing contralateral breast cancer risks in breast cancer survivors [15]. We have termed this novel application of reduced-dose radiotherapy for contralateral breast cancer prevention “Prophylactic Mammary Irradiation” (PMI) [15].

Although the number of pre-malignant target cells in the contralateral breast is not well defined, because the dependence of the required radiation dose on this number is logarithmic rather than linear [22], any realistic number of pre-malignant cells (e.g. up to several thousand) could be killed by a dose of around 20-25 Gy in 10 fractions [3]. If the PMI dose is chosen appropriately, the benefit in terms of cancer risk reduction for breast cancer survivors would be expected to significantly outweigh the small risks of radiation-induced cancer or other radiation-induced normal-tissue complications [15]. 

**Figure 1:**
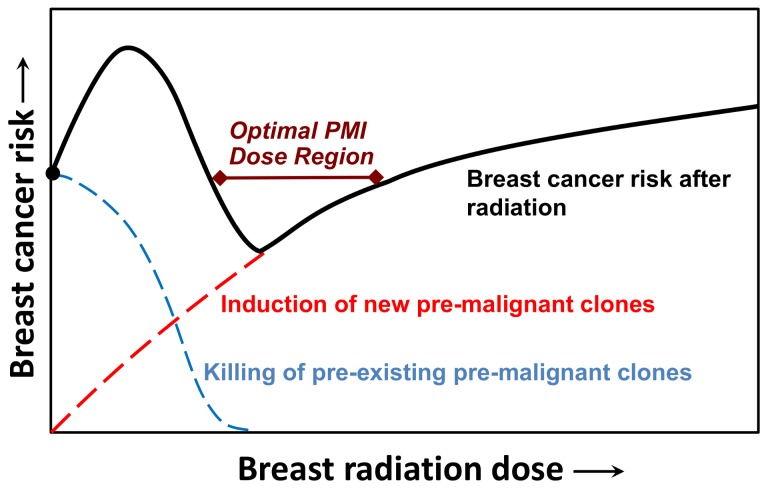
Schematic of radiation dose-effects on breast-cancer risk in the contralateral breast of breast cancer patients. The overall cancer risk is determined by a balance between killing of pre-existing pre-malignant cell clones vs. induction of new pre-malignant cell clones by radiation. The hypothesis underlying the present work is that there is a dose “window” at intermediate doses where killing of pre-existing pre-malignant clones dominates, thus reducing overall cancer risks.

Figure 1 shows a schematic of the expected overall cancer risk as a function of PMI dose, for a breast at high initial risk of a future cancer, i.e. containing a significant number of pre-existing pre-malignant cells. At low doses, only a few pre-existing pre-malignant cells will be killed and the mutagenic effects of the radiation may cause a net increase in cancer risk. As the dose increases, killing the pre-existing pre-malignant cells will dominate, and the overall cancer risk is expected to decrease. Finally, when all the pre-existing pre-malignant cells have been killed, still further radiation doses would be expected to increase the risks of cancer and/or normal tissue complications. 

Thus, PMI of the whole contralateral breast, performed at the same time as standard post-lumpectomy radiotherapy to the ipsilateral breast, and with an optimally chosen intermediate radiation dose, might be a potentially breast-conserving option to significantly decrease the high second cancer risks in the contralateral breast of long-term breast cancer survivors [15]. Because radiation-induced cell killing is likely to be independent of estrogen receptor status, this approach would be expected to be equally effective for estrogen receptor positive or negative tumors. It is emphasized that this concept would only be useful for an organ at high risk (i.e. containing large numbers of pre-malignant cells), and for an organ where the radiation-induced cancer risk is low. The contralateral breast of a breast-cancer survivor over the age of about 50 meets these criteria.

Here we have tested the PMI hypothesis in an animal model for high-risk breast cancer – the FVB/N-Tg(MMTV-PyVT)634Mul/J (abbreviated as MMTV-PyVT) female transgenic mouse [23]. Our goal is to investigate whether the predicted pattern of cancer risk with increasing PMI dose, as illustrated in Figure 1, holds for a high-risk mammary gland. MMTV-PyVT mice develop multiple mammary tumors at an early age (typically <100 days) due to the polyoma virus T-antigen (PyVT) driven by the mouse mammary tumor virus (MMTV) promoter. We investigated the effects of various doses of mammary irradiation (up to 16 Gy delivered in 4 Gy fractions) on tumor development in these mice, and show that the expected breast cancer risk pattern with increased dose (Figure 1) was indeed apparent: specifically, a small increase in cancer risk at low radiation doses, followed by a decrease in cancer risk at PMI-relevant doses, with the mammary tumor incidence rate reduced by a factor of about three.

## Materials and Methods

This study was approved by Columbia University IACUC, under Animal Care Protocol AC-AAAD3951, and all mouse work was performed in accordance with IACUC guidelines. Mammary tumor incidence rates were measured as a function of radiation dose in: 1) irradiated mammary glands, 2) shielded mammary glands exposed only to low doses of scattered radiation, and 3) unexposed control animals.

### Transgenic Mice

To conduct proof of principle experiments regarding the PMI hypothesis, we selected the FVB/N-Tg(MMTV-PyVT)634Mul/J female mouse (MMTV-PyVT) [23], from the Jackson Laboratory. In this mouse strain, an oncogene derived from the polyoma virus is expressed under hormonal regulation in mammary gland tissues, driven by the mammary tumor virus (MMTV) promoter [23]. Female transgenic mice of this strain begin to develop palpable mammary tumors in early adulthood (the median age of tumor onset is 66 days), and all the mice will eventually develop tumors [24]. Therefore, the progression from pre-malignant to malignant breast disease, which occurs in breast cancer patients, is mimicked by this mouse model, but in a more aggressive and rapid manner. This allows proof of principle testing of PMI to be conducted *in vivo*, minimizing mouse numbers and follow-up time. 

### Mouse Irradiation Geometry

**Figure 2:**
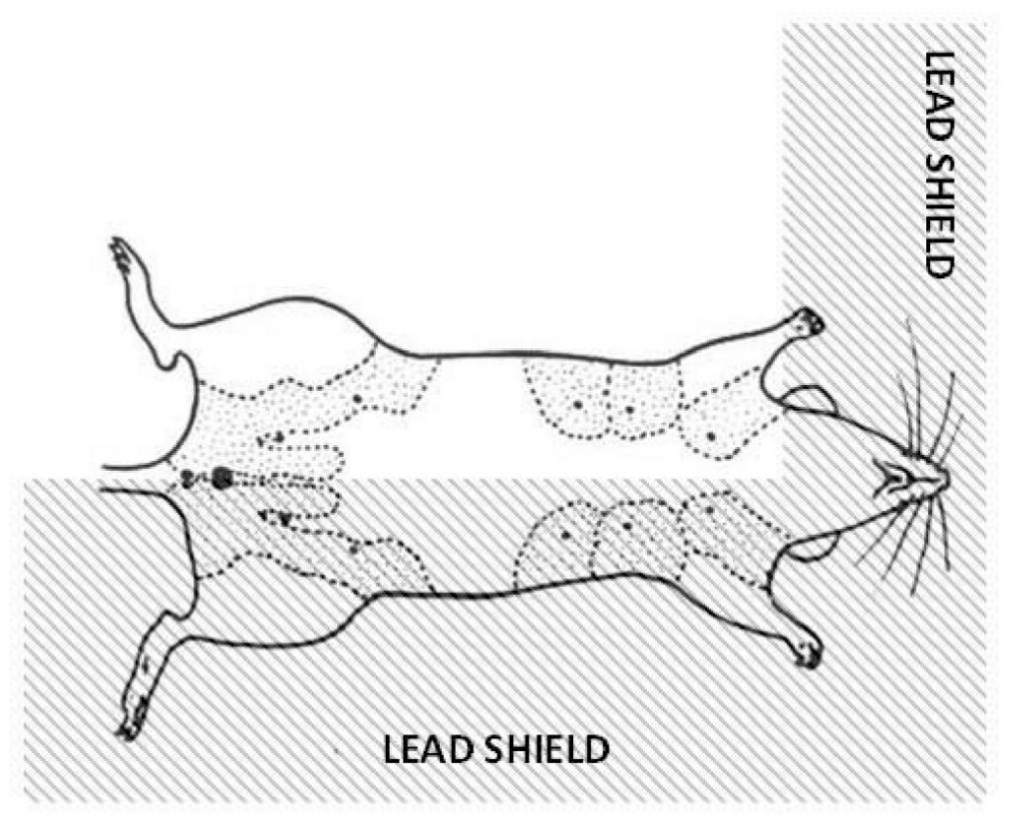
Shielding geometry for mouse mammary irradiations. Schematic ventral view of a partially lead-shielded mouse, with mammary glands outlined by dotted lines. Unshielded mammary glands on one side of the mouse received the full radiation dose, while the mammary glands on the lead-shielded side received a much lower dose – about 6% of the unshielded dose.

The near hemi-body irradiation geometry used here is illustrated in Figure 2. One side of each mouse, as well as the head, was shielded with 12.7 mm of lead. This geometry allows most of the body to be protected, exposing only one side of the thorax and abdomen and five mammary glands. The left side was unshielded in half of the mice, and the right side in the other half. This partial-body irradiation geometry has been described in more detail elsewhere [25]; the main radiosensitive organs, such as the bone marrow, lungs, intestinal tract, and ovaries, are protected on at least one side of the body, which allows the irradiated animals to remain in good health for long enough after irradiation to develop mammary tumors. In particular, allowing one ovary to be shielded from high-dose radiation exposure was designed to minimize indirect effects of radiation on mammary carcinogenesis through effects on ovarian hormone production.

### Choice of Radiation Doses

One hundred female MMTV-PyVT mice, divided into 5 groups of 20 mice each, were used. The mice were between 41 and 43 days old at the start of irradiation. Each group respectively received total x-ray doses to the unshielded mammary glands of 0, 4, 8, 12 and 16 Gy, delivered in 4-Gy fractions. The maximum number of fractions per day was two, 6 hours apart. In summary, the unexposed control mice received a single sham-irradiation, the 4 Gy exposed mice received a single irradiation with 4 Gy, and the 8 Gy exposed mice received two 4 Gy irradiations 6 hours apart on the same day. The 12 Gy exposed mice received two 4 Gy irradiations 6 hours apart on the first day, and a third 4 Gy irradiation on the second day. The 16 Gy exposed mice received two 4 Gy irradiations 6 hours apart on the first day, and two more 4 Gy irradiations 6 hours apart on the second day. 

The two highest doses (12 and 16 Gy in 4-Gy fractions) were designed to be in the range that would sterilize most or all of the pre-existing pre-malignant and/or early malignant cells in each mouse mammary gland (roughly estimated to number several thousand in female mice [26,27]), which are the likely progenitors of the mammary tumors. These two highest doses (12 and 16 Gy in 4-Gy fractions), thus designed to be the putative PMI doses, are also approximately equivalent in terms of cell killing to the originally proposed [14] clinical PMI doses of 17 to 20 Gy in 10 fractions. 

### Mouse Dosimetry

The x-ray irradiations were carried out using an XRAD-320 irradiator (Precision X ray, North Branford, CT) operated at 320 kVp and 12.5 mA, with a filter composed of 1.5 mm Al, 0.25 mm Cu and 0.75 mm Sn. The dose rate was 0.86 Gy/min. Immediately prior to irradiation, the mice were anesthetized with a 0.2 ml intraperitoneal injection of a solution of 10 mg/ml ketamine and 1 mg/ml xylazine. Control mice, receiving 0 Gy, were anesthetized and sham-irradiated.

Doses were measured using microMOSFET [28] solid-state dosimeters (Best Medical, Ottawa, Ontario, Canada) inserted into a tissue-equivalent mouse phantom. Due to x-ray scatter, the shielded contralateral mammary glands received an average dose of approximately 6% of the corresponding doses to the unshielded mammary glands. This ratio of doses in the shielded *vs.* unshielded mammary glands is similar to the ratio of doses in the contralateral *vs.* the irradiated breast in human breast radiotherapy [29].

### Mouse Carcinogenesis Assay

The mice were maintained with 50 mg/kg amoxicillin in the drinking water, and their health and body weights were monitored regularly. After irradiation, the mouse mammary glands were palpated at 2-3 day intervals. Appearance times and the sizes of mammary tumors were recorded. Mice were sacrificed according to Columbia University animal care guidelines when they met either of the following criteria: tumor diameter >20 mm, tumor size exceeding 10% of body weight, tumor ulceration, debilitating diarrhea, progressive dermatitis, rough hair coat, hunched posture, lethargy or persistent recumbence, coughing, labored breathing, neurologic signs (e.g. circling, head pressing, seizuring), bleeding from any orifice, self-induced trauma, and any condition interfering with eating or drinking (e.g., difficulty with ambulation). All mice eventually developed mammary tumors, and most had multiple tumors. The first tumor to present was invariably the one which caused the mouse to be sacrificed when it grew to a large size, and tumors which appeared later were still small at the time of sacrifice. Consequently, the incidence rate of the first detected tumor was selected as the most relevant endpoint in this study, and these data are presented here. 

### Statistical Analysis

The goal of this study was to assess how radiation dose affected the cumulative incidence rate (per mouse-day at risk) of the first detected palpable mammary tumors. This was done by comparing the cumulative tumor incidence rates in each radiation dosage group, on each mouse side (unshielded or shielded), with the rate in the unirradiated control group, using Poisson regression, as implemented by the WinPepi software package [30]. The data were consistent with Poisson assumptions, as suggested by the strong correlation (0.98) between standardized residuals and normal scores, and by the p-value (0.14) for the score test for the total model. 

Cumulative tumor incidence rates on different mouse sides were also compared within each radiation dosage group, using the 2-tailed mid-P approach [30] with Bonferroni correction for multiple pairwise comparisons. The 95% confidence intervals presented here for the incidence rate ratios were produced by the mid-P approach, and were similar to those generated by the Poisson regression approach mentioned above, and also by Fisher’s exact test. This same mid-P approach was also used to estimate 95% confidence intervals for the cumulative tumor incidence rate for each mouse side in each radiation dosage group. 

The potential presence of a monotonic trend in tumor incidence rate as function of radiation dose was evaluated by the Poisson trend statistic (Eq. 3.12 in Ref. [31]). Temporal trends in the accumulation of mammary tumors in each radiation dosage group, and on each mouse side, were analyzed by the logrank test. 

All the data pertaining to the mice used in this study, including body weights, dates of irradiation, tumor detection and sacrifice, are available upon request.

## Results

Each irradiated mouse received a fractionated radiation dose (varying from 4 to 16 Gy in different mice) to the unshielded mammary glands, and a very much lower dose (<1 Gy) to the shielded glands (0.2-0.9 Gy) caused by x-ray scatter. As seen in earlier studies of radiation-induced mammary cancer [32], the temporal kinetics of mammary tumor appearance showed no significant dose dependence: among the different dose groups (including the zero-dose and the scatter-dose groups) the median times of first detectable tumor appearance ranged only from 34.0 to 37.5 days post irradiation (or sham irradiation).

**Figure 3:**
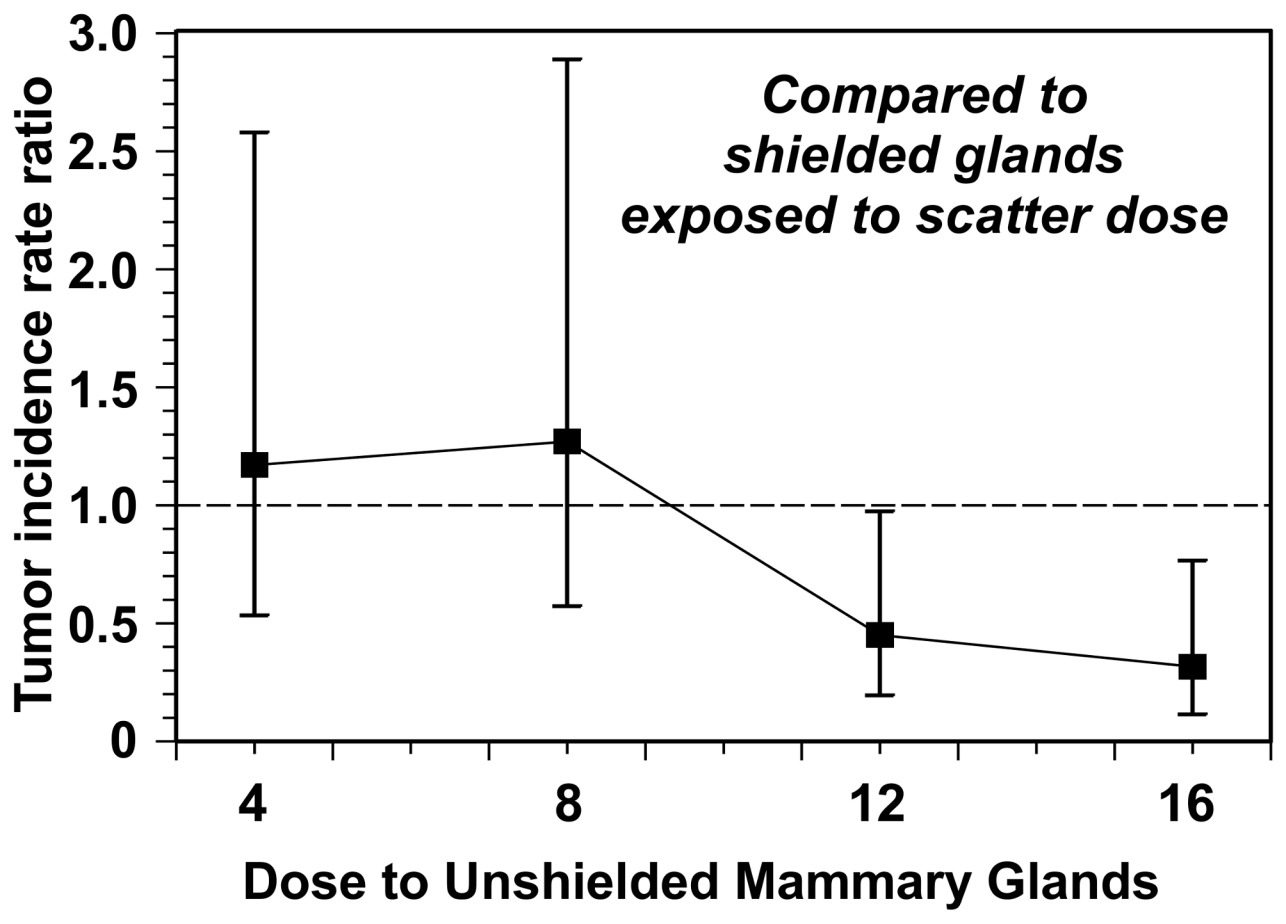
Tumor incidence rate ratios for tumors in unshielded irradiated mammary glands, compared to lead-shielded glands. The doses which were designed to be potentially relevant for PMI are 12 and 16 Gy. The comparison here is with lead-shielded contralateral mammary glands that were exposed only to low scatter doses – about 6% of the unshielded dose. Error bars represent 95% CIs estimated using the mid-P approach [30].

By contrast, the cumulative tumor-incidence rates (per mouse-day at risk) showed a clear dose response. Both relative to the zero dose controls, and relative to the corresponding mammary glands that received only scattered doses, tumor incidence rates initially increased with increasing dose to the irradiated mammary gland, but then decreased at higher doses to below the background levels. These results are summarized in Tables 1 and 2 and Figure 3.

**Table 1:** Mammary tumor incidence rates as a function of radiation dose, for zero dose controls, lead-shielded glands, and unshielded irradiated glands.

**Category**	**Dose (Gy)**	**# of dose fractions**	**Tumor incidence rate (per 1,000 mouse-days)**	
			**Rate**	95% CI*
**Zero dose controls**	0	1	14.2	9.4, 20.6
**Shielded glands**	0.23	1	14.2	7.7, 24.0
	0.46	2	12.0	6.3, 20.9
	0.69	3	22.4	14.1, 33.9
	0.92	4	22.1	13.7, 33.9
**Unshielded irradiated glands**	4	1	16.5	9.4, 27.0
	8	2	15.3	8.7, 25.1
	12 (PMI dose)	3	10.2	4.9, 18.5
	16 (PMI dose)	4	7.0	2.8, 14.5

The last two rows refer to glands irradiated at the doses which were designed to be relevant for PMI (12 and 16 Gy).

* The 95% CIs were estimated using the mid-P approach [30].

**Table 2:** Mammary tumor incidence rate ratios.

**Dose to unshielded irradiated glands (Gy)**	**Tumor incidence rate ratio vs. shielded scatter dose**			**Tumor incidence rate ratio vs. zero dose controls**		
	**Ratio**	95% CI*	p-value*	**Ratio**	95% CI*	p-value**
4	1.17	0.53,2.58	0.70	1.16	0.66, 1.90	>0.50
8	1.27	0.57, 2.89	0.56	1.09	0.61, 1.77	>0.50
12 (PMI dose)	0.45	0.20, 0.97	0.04	0.71	0.35, 1.30	>0.50
16 (PMI dose)	0.32	0.11, 0.77	0.009	0.49	0.20, 1.02	0.44

Shown are rates in unshielded irradiated mammary glands, compared with lead-shielded mammary glands that were exposed only to low scatter doses, and also compared with zero-dose controls. The last two rows refer to glands irradiated at the doses which were designed to be relevant for PMI (12 and 16 Gy).

^*^ The 95% CIs and 2-tailed p-values were estimated using the mid-P approach [30].

^**^ Bonferroni correction applied to adjust for multiple comparisons.

We focus here on comparisons between tumor incidence rates in the PMI-irradiated mammary glands and in the corresponding shielded mammary glands; as described above, this is designed to model a comparison of cancer rates in a human contralateral breast exposed to PMI vs. cancer rates in the same breast receiving a typical scatter dose after standard radiotherapy to the ipsilateral breast. At the doses which were designed to be relevant for PMI (12 Gy and 16 Gy, see above) the tumor incidence rates in the unshielded PMI-exposed glands were significantly lower than those in the shielded glands: the rate ratios between the PMI-exposed glands and the shielded glands were 0.45 (95% CI: 0.20-0.98, 2-tailed p value: 0.04) at 12 Gy and 0.32 (95% CI: 0.12-0.77, 2-tailed p value: 0.009) at 16 Gy (Table 2). 

These same patterns were apparent for the tumor rates in the PMI-exposed glands relative to zero-dose controls (Table 2). Although here statistical significance was not reached for any dosage group alone, the Poisson trend statistic [32] suggested a highly significant (p = 0.00012) decreasing trend in tumor incidence rate over the dose range from 4 Gy to 16 Gy. 

## Discussion

The measured dose response patterns (Tables 1-2, and Figure 3) are consistent with the expectations underlying the PMI hypothesis schematized in Figure 1. Specifically, at low doses there is an increase in radiation-induced breast cancer, presumably because few pre-existing pre-malignant cells are killed, and more new ones are induced. However, at the doses designed to be in the optimal PMI dose region (12 and 16 Gy), the overall breast cancer incidence rate was lower than in the controls, as predicted. Specifically, the optimal PMI dose in this study (16 Gy in 4 fractions) reduced the high background mammary tumor incidence rate by a factor of about three.

While there are similarities, there are of course differences between the high-risk animal breast cancer model used here and the contralateral breast in a breast cancer patient. Firstly, the mice were irradiated at an earlier time of life (early puberty) compared to most women (who develop breast cancer at a median age ~60): In fact both for rodents [33] and humans [16,18], radiation-induced breast cancer risks fall off sharply for age at exposure in middle age. Younger mice were used in this validation study explicitly to enhance any potential radiation-induced carcinogenic effects: that the prophylactic effects at PMI doses dominated these carcinogenic effects even in a young mammary gland (where these carcinogenic effects are maximal) suggests that in older mammary glands the cancer risk reduction from PMI may be even more pronounced. 

Secondly, it is unclear whether the relevant cells in the mouse model should be considered as pre-malignant or fully malignant, or perhaps a combination of these; this should not, however, affect the dose-effect patterns seen here, as killing of either would be expected to reduce cancer risk – and indeed the target cells for contralateral breast cancer in humans are also likely to be heterogeneous in their development. 

With these caveats, the results shown here are consistent with the predicted pattern of cancer risks in the PMI dose range. Specifically, we have provided proof-of-principle experimental confirmation that there is likely to be a “window of opportunity” in PMI dose (schematized in Figure 1) situated between low doses, where radiation-induced cancer is expected to dominate, and high doses, where both radiation-induced carcinogenesis and/or radiation-induced normal tissue damage will dominate: In this PMI intermediate-dose window the potential to inactivate essentially all the pre-existing pre-malignant cells in the high-risk breast is expected to dominate. This conclusion is consistent with recent evidence from a randomized control trial comparing intraoperative electron radiotherapy with conventional whole-breast external beam radiotherapy [34]. The trial showed that whole-breast irradiation was more effective at preventing new ipsilateral breast tumors, presumably because it kills pre-malignant cells in the entire breast, whereas intraoperative electron radiotherapy, which generates a cytotoxic dose mainly close to the tumor, presumably could not eliminate more distant pre-malignant breast cells.

Contralateral whole breast PMI may thus have promise as a spatially-targeted breast-conserving option for reducing the current high risk of contralateral second breast cancers in long-term breast cancer survivors. The potential efficacy of PMI is likely to be independent of estrogen receptor status: thus for estrogen-receptor positive primary tumors, PMI might optimally be used concomitantly with systemically delivered chemopreventive drugs such as tamoxifen or aromatase inhibitors, while for estrogen-receptor negative tumors, PMI might be used alone.

Should PMI prove successful, the potential impacts could be major. For example, there are currently more than 2.6 million breast cancer survivors alive in the US [1]: applying typical contralateral breast cancer incidence patterns [3] to this population suggests that about 160,000 of these patients will develop a contralateral breast cancer. If PMI reduced contralateral breast cancer incidence by 3-fold, as was the case in the rodent data shown here, approximately 100,000 breast cancer cases might be prevented.
